# A process evaluation of a peer education project to improve mental health literacy in secondary school students: study protocol

**DOI:** 10.1186/s12889-021-11921-3

**Published:** 2021-10-18

**Authors:** Emily Widnall, Steve Dodd, Ruth Simmonds, Helen Bohan, Abigail Russell, Mark Limmer, Judi Kidger

**Affiliations:** 1 Population Health Sciences, Bristol Medical School, University of Bristol, Canynge Hall, Bristol, BS8 2PL UK; 2 Lancaster University, Lancaster, UK; 3 Mental Health Foundation, London, UK; 4 College of Medicine & Health, University of Exeter, Exeter, UK

**Keywords:** Mental health, Peer education, Schools, Adolescents, Process evaluation

## Abstract

**Background:**

Emotional disorders in young people are increasing but studies have found that this age group do not always recognise the signs and symptoms of mental health problems in themselves or others. The Mental Health Foundation’s school-based Peer Education Project (PEP) has the potential to improve young people’s understanding of their own mental health at a critical developmental stage (early adolescence) using a peer teaching method. This study is a process evaluation to understand: the mechanisms through which PEP might improve young people’s mental health literacy, any challenges with delivery, how the project can be embedded within wider school life and how it can be improved to be of most benefit to the widest number of young people. We will also validate a bespoke mental health literacy questionnaire, and test the feasibility of using it to measure outcomes in preparation for a future study evaluating effectiveness.

**Methods:**

All schools recruited to the study will receive the PEP intervention. The process evaluation will be informed by realist evaluation approaches to build understanding regarding key mechanisms of change and the impact of different school contexts. The evaluation will test and revise an existing intervention logic model which has been developed in partnership with the Mental Health Foundation. Process evaluation data will be collected from newly recruited schools (*n* = 4) as well as current PEP user schools (*n* = 2) including training and lesson delivery observations, staff interviews and student focus groups. Baseline and follow-up data will be collected in all newly recruited intervention schools (*n* = 4) from all students in Year 7/8 (who receive the PEP) and recruited peer educators in Year 12 via a self-report survey.

**Discussion:**

This study will enable us to refine the logic model underpinning the peer education project and identify areas of the intervention that can be improved. Findings will also inform the design of a future effectiveness study which will test out the extent to which PEP improves mental health literacy.

## Background

Research suggests that up to 50% of mental health problems are established by age 14 [1]. Recent surveys of children and young people in the UK in 2017 and 2020 demonstrate an increase in disorders among 11–16-year-olds, from 12.6% having a probably mental disorder in 2017 to 17.6% identified as having a probable mental disorder in 2020 [2, 3]. A knowledge gap has been identified amongst young people regarding recognition of the signs and symptoms of mental health problems in themselves and others [4]. A lack of awareness of the signs of mental ill health can prevent timely help seeking [5], as can stigmatising attitudes towards mental health problems [6]. Mental health literacy has been conceptualised into four domains: 1) understanding how to obtain and maintain positive mental health; 2) understanding mental disorders and their treatments; 3) decreasing stigma related to mental disorders; and 4) enhancing help-seeking efficacy (knowing when and where to seek help) [7–9]. .Studies have highlighted low levels of mental health literacy among adolescents [10].

Schools are increasingly recognised as key to addressing the high prevalence of mental health difficulties among young people [11], particularly through whole-school, preventative approaches that focus on resilience and promotion of wellbeing [4, 12]. There is some evidence that school-based mental health literacy programmes can lead to improvements in knowledge and attitudes [13]. As young people often approach their peers for support with mental health concerns [4], higher levels of mental health literacy may increase the likelihood that young people signpost their friends and classmates effectively, as well as seeking help themselves, leading to improved outcomes [14].

Peer-delivered health education seeks to capitalise on young people’s tendency to turn to peers for support and advice, and has shown promise in fields such as sexual health [15], drug and alcohol misuse and smoking cessation [16]. However, there is still a scarcity of good quality evidence about the effectiveness of youth peers as health educators, particularly in mental health [17], with existing studies often deploying peer education methods because they are relatively cheap and wide-reaching, rather than because they have been established as effective. Models of peer education hypothesise that peers make effective health educators because (i) young people deem their peers more credible messengers of health information, (ii) peer education builds on existing social networks, (iii) peer educators become role models, influencing behaviour and opinions by modelling good practice [18] and iv) adolescents report they are more likely to seek advice from peers than adults at this developmental stage. However, there is limited research on peer-led initiatives related to mental health in schools [19].

The Mental Health Foundation’s Peer Education Project (PEP) aims to improve the mental health literacy of young people and decrease stigmatising attitudes towards mental health. Its focus is on behaviours that promote good mental health, and on risk and protective factors for mental health and help-seeking [20]. This study will contribute in-depth qualitative findings regarding the mechanisms by which the intervention may have an impact, providing perspectives from schools who have delivered the intervention for a number of years, as well as those who are new to the project. In addition, adaptations have had to be made to the intervention delivery during the current COVID 19 pandemic, and this evaluation will examine how acceptable these changes are to students and schools and which should be kept as permanent changes.

Systems theories suggest that both individual behaviour and the context in which these individuals operate are key to successful outcomes. The success of the Peer Education Project is therefore likely to depend on both individuals’ responses and on the wider school context. What works in one school or classroom, may not work in another. One approach that aims to understand both mechanisms of change and the impact of different contexts, particularly in health research, is realist evaluation [21]. Realist evaluation is a form of theory-driven evaluation based on realist philosophy which aims to advance understanding of why complex interventions work, how, who for and in what contexts [22]. The proposed study will therefore be informed by a realist approach to its evaluation in order to further understand the mechanisms of change underlying peer education and the impact of different school contexts.

### The PEP intervention

PEP is a school-based mental health education programme co-designed with Year 7 (age 11–12 years) and Year 12 students (age 16–17 years), with the primary aim of improving mental health literacy amongst students.

During the development of PEP, five consultation workshops were held with students in years 7 and 12 to develop the lesson content, which focused on: 1) Shared understanding and direction, 2) Learning outcomes and topic areas, 3) Lesson plans and outcomes and 4) Building competence and confidence for lesson delivery. Six consultation sessions were also carried out with school staff to consider the practicalities and timetabling of the project.

**Fig. 1:**
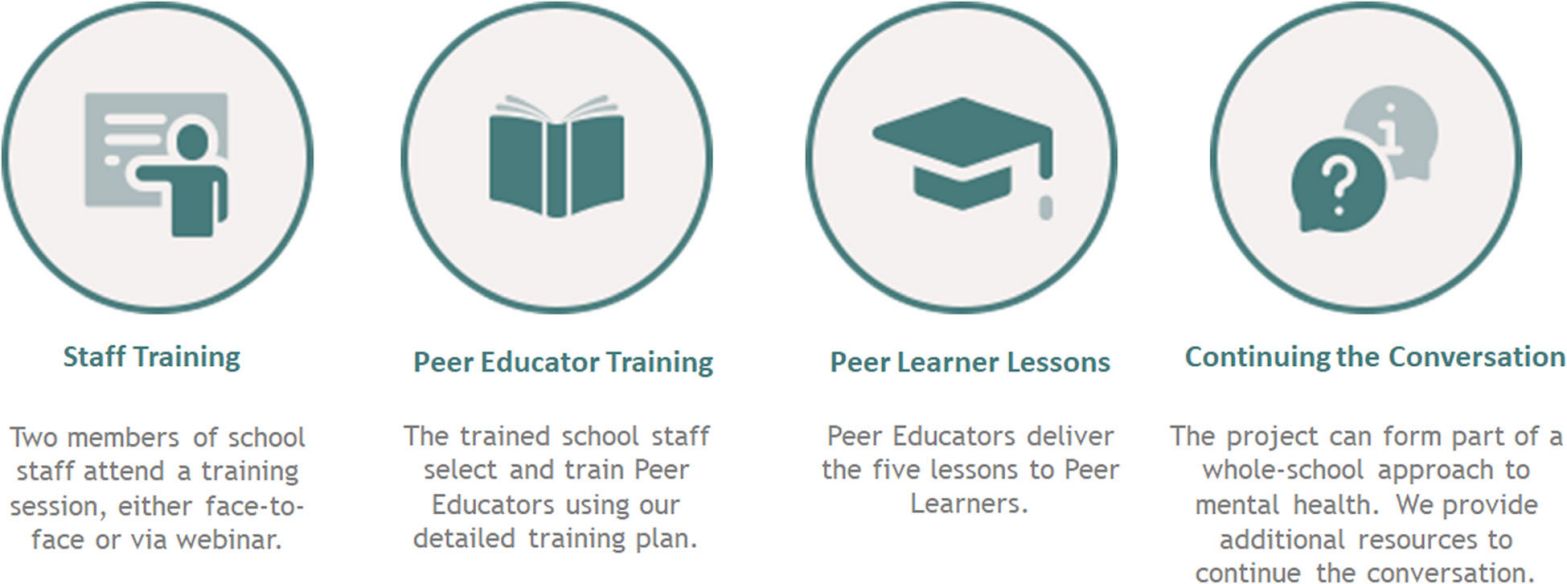
Peer education project overview

The programme consists of 4 stages, namely staff training, peer educator selection and training, lesson delivery and continuing the conversation (displayed in Fig. 1). The intervention offers an interactive five lesson mental health syllabus, covering basic mental health awareness, risk and protective factors for our mental health, ways to stay well, the importance of seeking help and how to support others. The syllabus is delivered by Peer Educators (older pupils) to Peer Learners (younger students), who are trained and supported by the school staff leading the project. Typically, peer educators are year 12 students, and peer learners are year 7 students.

The original theory of change underpinning the intervention when first developed by the Mental Health Foundation was based on four key components: i) it is peer-led, thereby making the messages potentially more relevant and acceptable to young people [15–17], ii) it is schools-based and delivered during Personal, Social, Health and Economic education lessons (PSHE), which are attended by the majority of young people iii) it is universal, thereby providing information to all young people, regardless of their current identified level of need [23], and iv) it is educational, and even short educational interventions have been shown to be effective in improving knowledge and attitudes around mental health, and reducing stigma [17, 24]. Knowledge and attitudes are important precursors of behaviour change, as they influence someone’s intent to perform a behaviour; behavioural intent is the strongest predictor of actual (observable) behaviour.

[20] The Mental Health Foundation have plans to expand the delivery of PEP considerably over the next few years, therefore the findings from this study will allow us to refine the intervention to maximise the public health benefits that could be achieved.

### Extending knowledge of PEP in the current study

In a previous evaluation of the programme [20], we have seen encouraging findings suggesting that PEP improved self-reported knowledge of mental health and confidence to discuss it. However, these evaluations have been limited by their lack of control groups, and the intervention content has been modified since this initial study. Before effectiveness can be more robustly tested, we want to ensure that all of the components of the programme are acceptable and feasible, and to understand more about how the intervention works in practice to inform development of a testable logic model. The key innovative feature of the intervention is the peer educators, so we want to understand how this element in particular is perceived by those involved, and any barriers that exist to its implementation. We will also explore how the intervention may lead to wider changes to the school culture around mental health support. In addition, we want to ensure that the programme is accessible to young people at most risk of developing mental health problems, particularly those in deprived areas. The present study will include schools in areas with different socio-economic profiles to help us understand more about how well the programme works, in what circumstances and for whom. The study seeks to understand what intervention components works best in different school contexts and to better understand what activities and contexts are essential for the intervention to be effective and which components are more flexible and could be adapted between schools.

In addition to understanding how the programme works, we will also test a novel mental health literacy questionnaire. Although there are a number of measures of various aspects of mental health literacy in the literature, it has been challenging to find one measure that includes all the domains in which we are interested (knowledge, attitudes, self-help strategies and help-seeking) and that is appropriate for 11–12-year-olds. We will develop and validate a survey that aims to measure all aspects of mental health literacy among this age group.

**Fig. 2:**
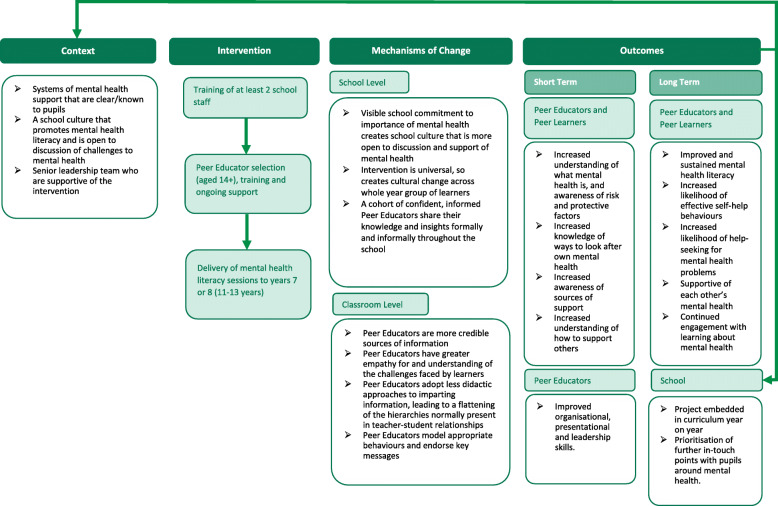
Peer education logic model

This study aims to:

i. Develop and test a mental health literacy measure for young teenagers
ii. Understand how the PEP intervention currently works in schools
iii. Understand the mechanisms of change within the PEP intervention
iv. Understand how the PEP intervention works in different school contexts
v. Identify areas in which the PEP intervention can be improved
vi. Test and revise the PEP logic model (Fig. 2)

## Methods/design

### Study design and sample

The study is a realist informed process evaluation of the Peer Education Project.

Initially, study recruitment consisted of inviting schools in two target areas (South West England and Lancashire) to participate. However, given COVID-19 disruptions to school life and subsequent challenges for schools to participate in additional activities, we will also invite schools that had already signed up to participate in the intervention via the Mental Health Foundation to participate. We will select those schools that are closest to the original target areas, ensuring a variety in free school meal status (above and below national average) as well as different school types (e.g. state schools and fee-paying schools).

Two ‘current user’ schools will be recruited who have previously run the PEP intervention, and up to four ‘new schools’ will be recruited; who have signed-up to run the PEP intervention during either the 2020/21 or 2021/22 academic year via the Mental Health Foundation.

Whole year groups of peer learners (Year 7/8 s) will participate in the intervention and a minimum of 3 peer educators per class of peer learners will be recruited. The number of peer educators and learners will therefore vary by school.

### Inclusion criteria

Secondary schools with a sixth form in England who are able to deliver the Peer Education Programme within the academic years of 2020–2022.

### Exclusion criteria

Sixth form only colleges, schools without a sixth form, pupil referral units and alternative provision/SEN schools will be excluded from the sampling frame.

#### Process evaluation data collection and outcomes

Observations, student focus groups and staff interviews will be carried out in both newly recruited and current user schools. A summary of these is detailed in Table 1. Student focus groups will cover young people’s experiences of delivering/receiving peer education lessons and views about what could be improved. Staff interviews will cover the perceived value of the PEP intervention, barriers and facilitators to its delivery as well as understanding how PEP fits in with the wider school context in terms of support for mental health.

**Table 1:** Summary of process evaluation

	Observations	Student focus groups	Staff interviews
New-user schools (*n* = 4)	Staff training (*n* = 1)	Peer educators (*n* = 4)	Pastoral Lead (*n* = 4)
	Peer educator training (*n* = 2)	Year 7/8 (*n* = 8)	Staff receiving/delivering training (*n* = 4)
	Lesson delivery (*n* = 2)		
Current user schools (*n* = 2)	Peer educator training (*n* = 1)	Peer educators (*n* = 2)	Pastoral Lead (*n* = 2)
	Lesson delivery (*n* = 2)	Year 7/8 (*n* = 4)	Staff receiving/delivering training (*n* = 2)
TOTAL	**8**	**18**	**12**

NB ‘n’ refers to number of data collection occasions

#### Survey data

##### Student and peer educator questionnaire

A self-report survey will be completed online in school time. Baseline and follow-up surveys will be completed by all trained peer educators and all year 7/8 students in the school. Peer educators will complete their baseline survey before they receive training and year 7/8 students will complete their survey before lesson delivery begins. Follow-up surveys will take place within 1 month of the final lesson.

The survey will measure four separate constructs: 1) student help-seeking using the General Help-Seeking Questionnaire (GHSQ) [25], 2) perceived peer support using the peer support subscale of the Sense of Belonging Scale [26], 3) student wellbeing using the short version of the Warwick–Edinburgh Mental Wellbeing Scale (SWEMWBS) [27] 4) Student mental health literacy, assessed by 10 true/false statements, which have been developed as part of this study and reflect the lesson content. This questionnaire will be validated as part of the study (detailed within data analysis).

#### Data analysis

##### Quantitative analysis

We will conduct descriptive analyses to investigate response rates and prevalence for the quantitative outcome measures. We will estimate the intraclass correlation (ICC) and the between-individual standard deviation for the main outcome measures to enable power calculations for the sample size that would be needed in a trial powered to detect intervention efficacy.

Additionally, we will test the reliability and validity of the newly developed mental health literacy questions. To test reliability, we will measure internal consistency using Cronbach’s alpha as well as carrying out test-retest reliability using ICC. We will test construct validity using related individual items from our other validated measures to assess strength of correlations.

##### Qualitative analysis

Qualitative data will be audio-recorded and transcribed verbatim. The qualitative data analysis software package NVivo 11 will be used to support analysis and data management. The analysis will take a realist evaluation perspective, seeking to understand how the intervention works, for whom and in what context. Context-mechanisms-outcomes configurations (CMOc) will be used for analysis [28, 29]. An initial framework of CMOcs will be developed by extracting relevant findings from related literature, and deduced from the initial logic model (Fig. 2). Data analysis will take a retroductive approach using both inductive and deductive logic to interrogate the causal factors that may have operated to produce outcomes. Interview and focus group data will first be deductively analysed to support and refute CMOCs and an inductive analytical approach will enable the exploration of new CMOCs. Peer educator, peer learner and staff interviews will be analysed together to enable us to understand how the three groups and interactions between them feed into the CMOcs. Nodes will be created in NVivo for each hypothesised CMOC and new child nodes will be added for any newly identified contextual conditions, mechanisms or outcomes. This approach will allow us to track the iterative refinement of the logic model.

## Discussion

This paper presents a protocol for the process evaluation of the Mental Health Foundation’s Peer Education Project; intended to improve young people’s understanding of mental health at a critical developmental stage using a peer teaching method.

Study findings will extend understanding of the mechanisms through which peer support models may impact upon student mental health and wellbeing. Findings will also provide valuable learning for the longer-term implementation of this particular intervention, including insight into ways in which it can be sustainable and create whole school change, and how it may be adapted from one school context to another. We will use the findings of the study to update and refine the intervention’s logic model, incorporating evidence about how the programme impacts Peer Educators and the wider school community, as well as the Peer Learners. We will also use the findings to improve the programme content, making adaptations to the way in which concepts are taught or instructions for delivery are given, based on detailed student feedback. This study will also validate a novel mental health literacy questionnaire which could be used to evaluate school-based mental health literacy interventions targeting early adolescence. .

## Data Availability

Not applicable.

## Abbreviations

PEP: Peer Education Project
MHF: Mental Health Foundation
NIHR: National Institute for Health Research
